# Improper Drainage and Disease

**Published:** 1884-04

**Authors:** 


### Editorial.

### IMPROPER DRAINAGE AND DISEASE.

In our recent report of the annual commencement of the Buffalo Medical College, reference was made to a paper on "Common Defects of House Drainage," read before the Alumni meeting by Dr. William F. Sheehan, Health Physician of the city of Rochester, N. Y. The paper was generally considered one of unusual interest, the presentation of the subject being materially aided by the exhibition of illustrations magnified and projected by means of the stereopticon. To hear the interesting remarks and see the apt and apparently corroborative illustrations, was to feel the impulse of agreement and of thankfulness that the etiology of a large class of diseases was clearly and incontrovertibly established. But, prompted though it may be by a constitutional predisposition to skepticism, we are yet led to ask, may this not be but another instance of the wish being father to the thought? The human mind, constantly striving to enlarge its field of well-defined and perfect vision, hails eagerly the faintest indications of the promise long deferred. We wonder not at this, neither do we murmur, if, as many times occurs, the evidence be unauthenticated and the welcome prove premature. On the contrary, by these repeated disappointments we are taught the inherent difficulties attending the elucidation of all scientific subjects. In a special degree should these difficulties be recognized in the study of the broad and complex subject of the etiology of disease, and here as elsewhere must we make full allowance for our natural proneness. Too often is conviction the offspring of predilection. Touching the question under discussion, we should remember that the arguments, positive and convincing as at first sight they may appear, are yet opposed by a vast amount of negative evidence. Negative evidence thus accumulated is justly entitled to serious consideration. And it should be borne in mind by those impressed with the seeming corroboration furnished by the illustration that these were purely diagramatic, so far as the essential point of filtration is concerned. The proposition that defective drainage and diseases bear to each other the relation of cause and effect may be true or it may not. It is not our present purpose to deny the possible truth. When, as seems not impossible, future additional observation and experiment shall have established the fact, it will, indeed, be a grand and positive advance in sanitary science. But meanwhile, we consider it opportune to warn against ill-considered judgment, prompted rather by a fixed though laudable desire than authorized by the present state of our limited and immature knowledge. While it is the obvious duty of every physician to promote the cause of medical science and to accept its discoveries so soon as they are such, in truth, no good has ever or can ever come, but rather much harm, from hasty acceptance of supposed facts. The medical man, no less than the worker in other fields of science, must be both progressive and conservative and know how to combine these qualities judiciously.

Relative to the subject under discussion, and to this we shall limit our direct criticism, we have already alluded to the fact that much negative evidence remains, apparently offering serious obstacles to its implicit and unquestioned acceptance. And since the positive argument has enlisted so many exponents and occupied so much of the thought of the profession, we feel it our duty to call attention to the fact that the matter as yet remains unproven—that the subject, like the bronze and silver shield in the legend, has two sides, which must receive equal and impartial attention, before we can venture to express an opinion or form a conviction. As yet the matter lacks the positive proof necessary to establish a scientific truth. Every scientific man recognizes the difficulty of these and kindred subjects; the arduous, repeated and guarded observations and experiments, as well as the patience and discrimination necessary to determine the most insignificant point in science.

The argument of the advocate is essentially expressed in the following syllogism:

First premise: Certain diseases are prevalent in a given locality. Second premise: In these localities drainage is many times, perhaps even generally, found to be defective. Conclusion: Ergo, the defective drainage is the cause of the prevalence of these diseases. With the laity of the present, as with the profession more particularly, though not exclusively, of the past, the phrase, *Post hoc ergo propter hoc*, is the almost universal and invariable channel of reasoning. How thoughtless and narrow-minded, say we of to-day; and yet the same phrase, slightly altered as follows, *Apud hoc ergo propter hoc*, would seem to be the motto of many of our medical brethren .Admitting this as correct or logical reasoning, what a plausible treatise might not be written, proving the causative relation existing between the enclosure of dwellings by picket fences and the peculiar susceptibility of the inhabitants to borborygmi.

But it is not our purpose to ridicule a theory which presents considerable supporting evidence and is daily becoming more probable. To establish the fact, however, it must be clearly and indubitably shown, not in singular cases but in the vast majority, that the coincidence is more than a simple coincidence, and in reality exhibits the relation of cause and effect. This is yet far from having been accomplished.

To do this it must first be explained how the supposed causes, having existed for an indefinite period, have remained so long inactive, and again become so after the disease has disappeared without any measures having been taken for their removal or mitigation. To say that the production of disease requires the coincidence of still another factor, such as, for example, a temporary susceptibility, may explain but fails to answer the vast amount of negative evidence and the apparent contradiction of certain well-established facts. As an example of the latter: In a family of six persons scarlatina attacks one of the six and consecutively the remaining five. If in these cases the cause was one of constant and uniform existence, should we not expect the attacks to have been more nearly contemporaneous? The reply that in this case the disease spread by infection, the original infection being from without, is probably correct. But can we doubt that in such a case many an aspiring advocate of the claims of defective drainage would have found this to exist, and have ascribed the disease to its vicious potentiality. Is defective drainage, measured by the standard of perfection laid down by experts, not the the rule rather than the exception? Rare are the dwellings where numerous violations of asserted sanitary laws, including defective drainage, cannot be found. Again, in the case of the supposed infection of well water, the remarkable filtering power of most earths must receive its due consideration; and it must be remembered that to find sodium-chloride and phosphate is not always to have shown the containing water poisonous. The various objections, more or less applicable and forcible, are found on every hand, and intrude themselves upon every thinking and impartial mind. And here, as elsewhere throughout the domain of science, their mission is to warn us, that while promoting and welcoming every positive gain in knowledge, it yet behooves the physician to guard vigilantly against rash conclusions and too implicit and uncritical acceptance of unproven *dicta.*

